# Inhomogeneities in the Catholyte Channel Limit the Upscaling of CO_2_ Flow Electrolysers

**DOI:** 10.1021/acssuschemeng.2c06129

**Published:** 2023-02-07

**Authors:** Joseph W. Blake, Vojtěch Konderla, Lorenz M. Baumgartner, David A. Vermaas, Johan T. Padding, J. W. Haverkort

**Affiliations:** † Department of Process and Energy, Delft University of Technology, Leeghwaterstraat 39, 2628 CBDelft, The Netherlands; ‡ Department of Chemical Engineering, Delft University of Technology, 2629 HZDelft, Netherlands

**Keywords:** CO_2_ reduction, electrolysis, gas diffusion electrode, scale up, variable catalyst loading, parasitic reactions

## Abstract

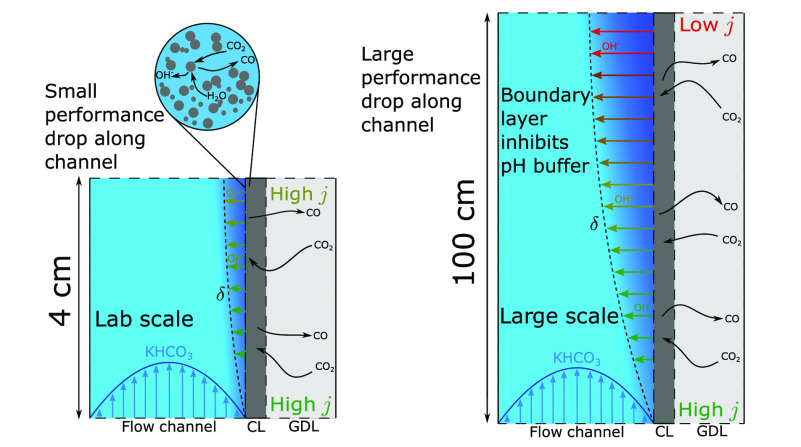

The use of gas diffusion electrodes that supply gaseous CO_2_ directly to the catalyst layer has greatly improved the performance of electrochemical CO_2_ conversion. However, reports of high current densities and Faradaic efficiencies primarily come from small lab scale electrolysers. Such electrolysers typically have a geometric area of 5 cm^2^, while an industrial electrolyser would require an area closer to 1 m^2^. The difference in scales means that many limitations that manifest only for larger electrolysers are not captured in lab scale setups. We develop a 2D computational model of both a lab scale and upscaled CO_2_ electrolyser to determine performance limitations at larger scales and how they compare to the performance limitations observed at the lab scale. We find that for the same current density larger electrolysers exhibit much greater reaction and local environment inhomogeneity. Increasing catalyst layer pH and widening concentration boundary layers of the KHCO_3_ buffer in the electrolyte channel lead to higher activation overpotential and increased parasitic loss of reactant CO_2_ to the electrolyte solution. We show that a variable catalyst loading along the direction of the flow channel may improve the economics of a large scale CO_2_ electrolyser.

## Abstract 2

The current scale of CO_2_ electrolysers is too small to be industrially feasible. We develop a computational model to determine the causes of electrolyser upscaling issues and their possible solutions.

## Introduction

CO_2_ from atmospheric or industrial sources can be electrochemically converted to valuable chemicals and fuels, leading to carbon neutral energy storage solutions and chemical feedstocks. The use of gas-diffusion electrodes (GDEs) allows the problems of low CO_2_ solubility and diffusivity in aqueous electrolytes to be minimised, leading to high current density lab scale electrolysers.^1^ However, the industrial realisation of this technology is hindered by a poor understanding of how these processes work in industrially relevant conditions. A rough benchmark of a minimum current density of 200 mA cm^–2^ has been reported for commercial feasibility,^2−4^ and this current density must be achieved at industrial scales. The typical lab scale cell has a geometric surface area around 5 cm^2^ but an industrial scale cell would be significantly larger: Verma et al. proposed a surface area of 400 cm^2^ per cell,^3^ while analyses that make comparisons to alkaline water electrolysis propose even surface areas from 1000 cm^2^ to 1 m^2^ and above.^5,6^

However, upscaling in the flow direction leads to issues that negatively affect the performance of larger electrolysers. The high pH gradients, which have little impact on short lab scale electrolysers, lead to excessive pH increase in the extended flow direction. This high pH induces a Nernstian potential shift and depletes CO_2_ through the carbonate equilibrium reactions, leading to reduced current density, Faradaic efficiency (FE), and reactant utilisation. However, the lack of experimental upscaling studies means the mechanisms behind these issues and the severity of their effects are poorly understood. In this paper we investigate the effect of scale by developing a 2D model of a typical lab scale CO_2_ electrolysis cell and compare it to models that extend the geometry in the flow direction to 1 m. We identify the causes and magnitude of the performance loss and provide strategies to minimise the issues.

## Theory

**Figure 1:**
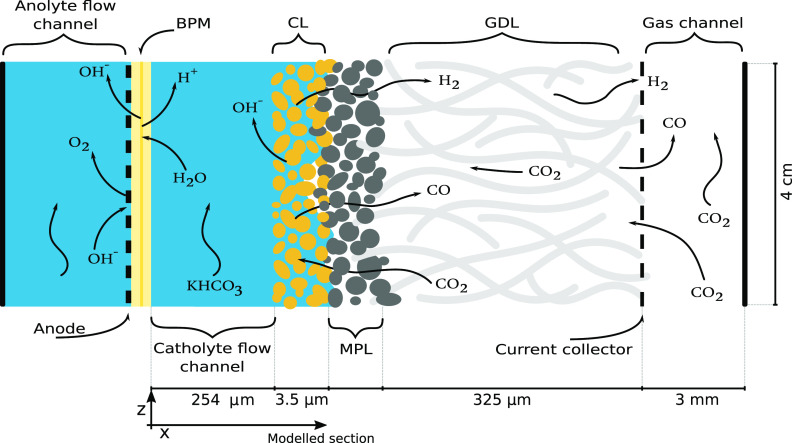
Diagram of a typical electrolysis cell (not to scale). From right to left: the gas channel, which supplies gas phase CO_2_; the gas-diffusion layer (GDL), a dry macroporous structure through which CO_2_ can permeate; the microporous layer (MPL), a thin hydrophobic layer that prevents electrolyte leakage into the gas-diffusion layer while allowing CO_2_ dissolution into the liquid phase; the catalyst layer (CL), in which liquid electrolyte and catalyst particles embedded in the porous structure facilitate the electrochemical reaction with the dissolved CO_2_; the catholyte flow channel, through which liquid electrolyte is supplied; a bipolar membrane (BPM), in which water dissociation supplies H^+^ and OH^–^ ions to the respective sides; the anode, on which the counter reaction is performed; and the anolyte flow channel.

Figure 1 shows a schematic diagram of an electrolyser setup^7,8^ for CO_2_ electrolysis flow cell with a liquid electrolyte channel. This channel is sometimes replaced with a membrane-electrode assembly (MEA),^9,10^ and similarly, it is not unusual for an anion-exchange membrane (AEM)^11−13^ to be used in place of the bipolar membrane (BPM) shown.^14−18^ Gaseous CO_2_ enters through the gas channel and passes through the macroporous gas diffusion layer (GDL) and microporous layer (MPL) to enter the liquid catholyte in the porous catalyst layer (CL), in which the heterogeneous electrochemical reaction takes place on the catalyst particles suspended in ionomer in the CL. The porous structures in the cell are modelled as macrohomogeneous domains characterised by porosity, ε, permeability, κ, and volumetric surface area, *a*_v_. While it is possible for the exact location of the gas–liquid interface to vary in the neighbourhood of the CL-MPL boundary when hydrophobic CLs are used,^19,20^ it is assumed in the model that the CL remains fully saturated with liquid electrolyte, while the MPL and GDL remain entirely dry at all times. This allows the combination of the GDL and MPL into one effective diffusion layer, a practice that is common in the determination of bulk properties of commercial GDLs supplied with preprinted MPLs.

### Reactions

The ionomer in the CL is considered to be impregnated with evenly distributed Ag catalyst particles that facilitate heterogeneous reactions, and due to the high Faradaic efficiency (FE) of Ag catalysts towards CO, we consider only the reduction of CO_2_ to CO
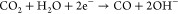
1and the competing hydrogen evolution reaction (HER),
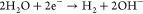
2While there is an argument for the HER pathways involving H^+^ and HCO_3_^–^ to be included, these pathways are prevalent in near-neutral pH environments,^21^ and previous modelling studies predict that the CL environment will be very basic at current densities greater than 100 mA cm^–2^^22^ and that this pH will only increase in the flow-wise direction.^23^ On the anode, the oxygen evolution reaction (OER),
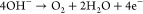
3takes place.

A major complication distinguishing CO_2_ electroreduction from similar technologies arises from the homogeneous reaction of CO_2_ with OH^–^ to form bicarbonate and carbonate. These reactions can take both acidic and basic pathways, neither of which can be neglected. The basic
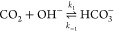
4
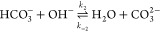
5and acidic
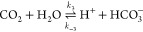
6
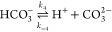
7reactions have the same ratios of CO_2_, HCO_3_^–^, and CO_3_^2–^ at equilibrium for a given pH due to their coupling in the water self-ionisation reaction
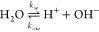
8Furthermore, the equilibrium constants, , for the acidic reactions are related to the basic reactions through *K*_3_ = *K*_1_*K*_*w*_ and *K*_4_ = *K*_2_*K*_*w*_. However, the reactions have different kinetic rates and as such are each modelled individually.

The reaction rates per unit volume for species *i* in heterogeneous reaction *r* are given by
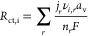
9where *j*_*r*_ is the local current density of reaction *r*, ν_*i*,*r*_ is the stoichiometric coefficient of species *i* in reaction *r*, *a*_v_ is the volumetric surface area, *n*_*r*_ is the number of electrons transferred in reaction *r*, and *F* is Faraday’s constant. The local current densities at the anode and cathode are determined using the anodic and cathodic branches of the Butler–Volmer equation, respectively, in the forms of
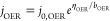
10
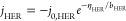
11
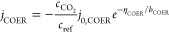
12where *j*_0,*r*_ are the exchange current densities, *b*_*r*_ are the Tafel slopes, and η_*r*_ are the activation overpotentials. The COER equation includes the concentration dependence on CO_2_ with respect to a reference concentration *c*_ref_, but the other two reactions are assumed to have a constant reactant source of water, and as such no explicit concentration dependence is necessary. In this case, *c*_ref_ should refer to the equilibrium concentration, but in some derivations of kinetics a different reference concentration, such as 1 M,^22^ is used, leading to a correspondingly altered derived value of exchange current density. The Tafel slopes are given by
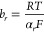
13where α_*r*_ are the charge transfer coefficients for their respective Tafel branches and *R* is the ideal gas constant. The activation overpotentials η_*r*_ are defined as
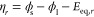
14where ϕ_*s*_ and ϕ_*l*_ are the electrode and electrolyte potentials, respectively, and *E*_eq,*r*_ is the equilibrium potential of reaction *r*, corrected for the local pH through the a simplified Nernst equation assuming water and solute activities are near unity,
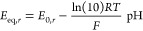
15Nesbitt et al. noted that this form of the Nernst equation is determined using the assumption of an acidic or near neutral electrolyte in which the reaction equation reads
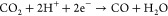
16and the rate-determining step is the second protonation by H_2_O,^24^ but it is often assumed without verification that this remains the rate-determining step for the reaction pathway in alkaline media, eq 1. Noting that the pH dependency in eq 15 is roughly −0.0591 pH, they give simplified Nernst equations for acidic/neutral electrolytes and alkaline electrolytes,
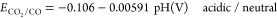
17
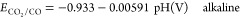
18and note a common misnomer in literature in which eq 1 is associated with eq 17. Reaction kinetics determined under these assumptions are frequently used in literature^22^ and while the resulting current density-potential curves may give results similar to experiments, further experimental verification of the reaction mechanism in alkaline media and subsequent recalculation of kinetic parameters are necessary to describe the dependence of the reaction on local activities. The reaction rates from the homogeneous chemical reactions, *R*_H,*i*_, are given by
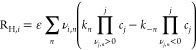
19where ν_*i*,*n*_ are the stoichiometric coefficients of species *i* in homogeneous reaction *n*, and *k*_±*n*_ are the forward and backward reaction rates of reaction *n*.

### Transport

The gas phase consists of 99.99% pure CO_2_ at the inlet (0.01% N_2_) with additional CO and H_2_ components at the outlet. Transport in the gas phase from the gas channel through the GDL and MPL is modelled with Darcy’s law and a mixture averaged diffusion model approximation requiring only binary diffusion coefficients.^25,26^ Darcy’s law reads
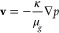
20where **v** is the gas velocity field, κ is the permeability of the porous medium, μ_*g*_ is the dynamic viscosity of the gas, and *p* is the pressure. The flow is assumed incompressible and density variations are neglected. The diffusive mass flux is assumed to be proportional to the mole fraction gradient through
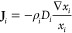
21where **J**_*i*_ is the mass flux of species *i* relative to the average fluid velocity **v**, ρ_*i*_ is the species density, *D*_*i*_ is the porosity and finite pore size corrected mixture-averaged diffusion coefficient, and *x*_*i*_ is the mole fraction. The mixture-averaged diffusion coefficients are determined from the binary diffusion coefficients in
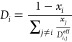
22where *D*_*i*_^eff^ are the effective binary diffusion coefficients corrected for porosity and tortuosity through the Bruggeman correlation,^27^
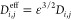
23Gas transport is usually slightly poorer in the MPL than in the GDL because MPLs typically have lower permeabilities and porosities than GDLs, but this reduction is small compared to the effect that electrolyte intrusion would have on the GDL. Regardless, it is common for manufacturers to supply GDLs with MPLs already applied, and provide the averaged values of transport properties of the final bilayer structure rather than of the individual components. We thus elect to model the GDL and MPL as one numerical domain with uniform porosity and permeability.

In the liquid phase there is bulk flow in the electrolyte channel, but the effect of change in permeability on transition into the porous CL is sufficient that flow in the CL can be neglected.^28^ Transport of species is modelled by assuming that concentrations remain small enough to be treated as a dilute solution and using the Nernst–Planck equation:
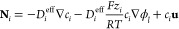
24where **u** is the liquid velocity and *z*_*i*_ is the species charge number. Species conservation in the steady state is ensured by
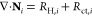
25and electroneutrality is ensured by
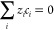
26allowing the transport equations in eqs 24 and 25 to be solved for all but one of the species, with the final concentration determined through the electroneutrality condition in eq 26. The local liquid velocity **u** is determined assuming a Poiseuille flow profile,
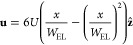
27where *U* is the average velocity. A similar method is used for the inlet gas velocity but with *W*_GAS_ instead of *W*_EL_, and *U*_*g*_ prescribed based on the target single pass conversion. At the liquid–gas phase interface, the flux is described by
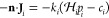
28where  is the Henry constant for species *i*, *p*_*i*_ is the partial pressure of that species and *k*_*i*_ is a characteristic cross phase mass transfer coefficient determined for CO_2_ adsorption into the CL ionomer by Kas et al.^23^ The Henry constant is further corrected for ionic concentrations within the electrolyte (see eq 6 in the SI) following the Sechenov equation.^29−31^ The solubilities of the remaining gas species are around 2 orders of magnitude lower than that of CO_2_, so their Henry constants are negligible and they cannot enter the CL, only exit. CO_2_ entering the liquid phase causes  to progressively decrease along the flow channel, subsequently decreasing the flux in eq 28.

## Computational Model

The equations for the liquid phase are altered slightly to yield approximate expressions that prove significantly easier to resolve computationally. The homogeneous reactions are problematic due to the extreme magnitudes of the rate coefficients, so we use the assumption that the local environment will be alkaline in the vicinity of the cathodic reaction and acidic in the vicinity of the BPM to selectively neglect the alkaline pathways, *K*_1_ and *K*_2_, in the BPM half of the electrolyte channel and the acidic pathways, *K*_3_ and *K*_4_, in the cathodic half of the channel. Due to the coupling of their equilibrium constants we are assured that there is no significant discontinuity in net reaction rates at this halfway point so long as the concentration boundary layers do not extend further than half of the channel width. Furthermore, for numerical stability we adopt a logarithmic form of the transport equations in the liquid,
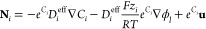
29where . *C*_*i*_ can now take any real value and the relation ensures that *c*_*i*_ is strictly positive. This entirely precludes the possibility of negative concentrations in the solution, which is otherwise a common computational issue in systems with high reaction rates and reactant depletion. Another limitation is the computational cost of the effect of local ionic strength variation on ϕ_*l*_ in eq 24. Extremely high homogeneous reaction rate coefficients lead to high species concentration sensitivities, even in regions with smooth solutions, leading to stiffness in the system. However, we can do little more to alleviate this without sacrificing significant detail in the homogeneous reactions. An approximation is made to remove the dependence of the migration term on concentration and instead use an iterative nonuniform electrolyte conductivity, given by
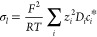
30where *c*_i_^*^ is the average concentration in the previous iteration of the numerical solver. This effectively decouples ϕ_*l*_ from eq 29 by determining it from Ohm’s law,
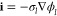
31and the state of the previous iteration, and allowing eq 29 to solve for *C*_*i*_ without simultaneously solving for ϕ_*l*_.

Despite these simplifications, the scale of the system is still too large for reasonable computation. To this end, the cell model is decomposed into the subcells shown in Figure 3. We take one initial short (4 cm) basis cell and find a stationary solution, and then solve a second subcell with the downstream outlet of the previous subcell used as the inlet condition. This approximation requires that the flow be sufficiently fast that transport against the flow direction can be neglected. This condition can be written as a necessarily high bulk Péclet number, the ratio of advective to diffusive transport rates. While there is no flow at the channel walls and in the CL, these regions are fully within the concentration boundary layer and the flow-wise concentration gradient is too small for diffusion to play a non-negligible role. Instead the flow wise variation in the CL is almost entirely determined by the concentration development in the adjacent electrolyte channel and in the gas phase, in what amounts to a locally 1D model, which is to be expected of a domain whose in plane dimension is 4 orders of magnitude greater than the through plane dimension. In the 4 cm model with no subcell decomposition it was already found that in plane diffusion was negligible in the CL, and it is expected that it would become even less important when the length scale is increased.

**Figure 2:**
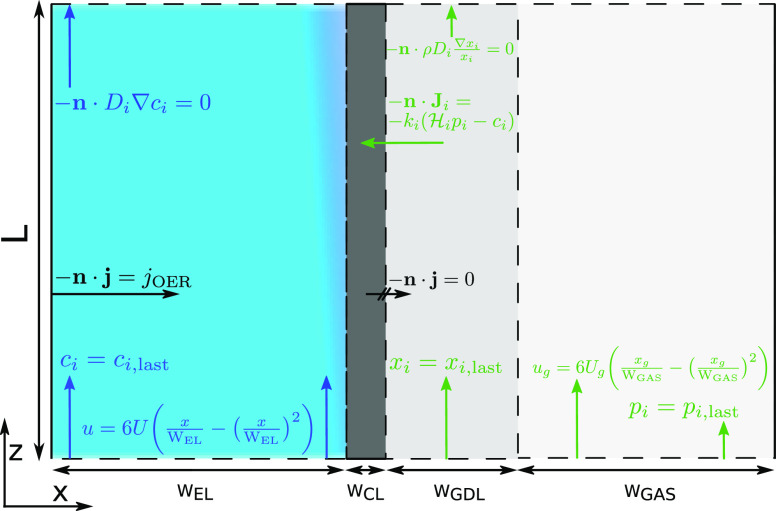
Schematic of the model equations and domains. The *z*-axis follows the flow along the catholyte channel of length *L*. The *x*-axis passes through the catholyte channel of width *W*_EL_, the CL of width *W*_CL_, the GDL of width *W*_GDL_, and into the gas channel of width *W*_GAS_. In black, the electrolyte current boundary conditions denote the anodic current source on the left and insulating condition on the right. In the liquid phase, blue equations denote the inlet condition on concentration, the open boundary outlet condition, and the Poiseuille flow velocity distribution in eq 27. In the gas phase, green equations denote the inlet conditions on partial pressures and mole fractions, the open boundary outlet condition, the liquid–gas mass transfer rate in eq 28, and the initial Poiseuille gas velocity distribution in the gas channel, where *x*_*g*_ denotes the coordinate within the gas channel and *U*_*g*_ is the initial average gas velocity for the simulation.

**Figure 3:**
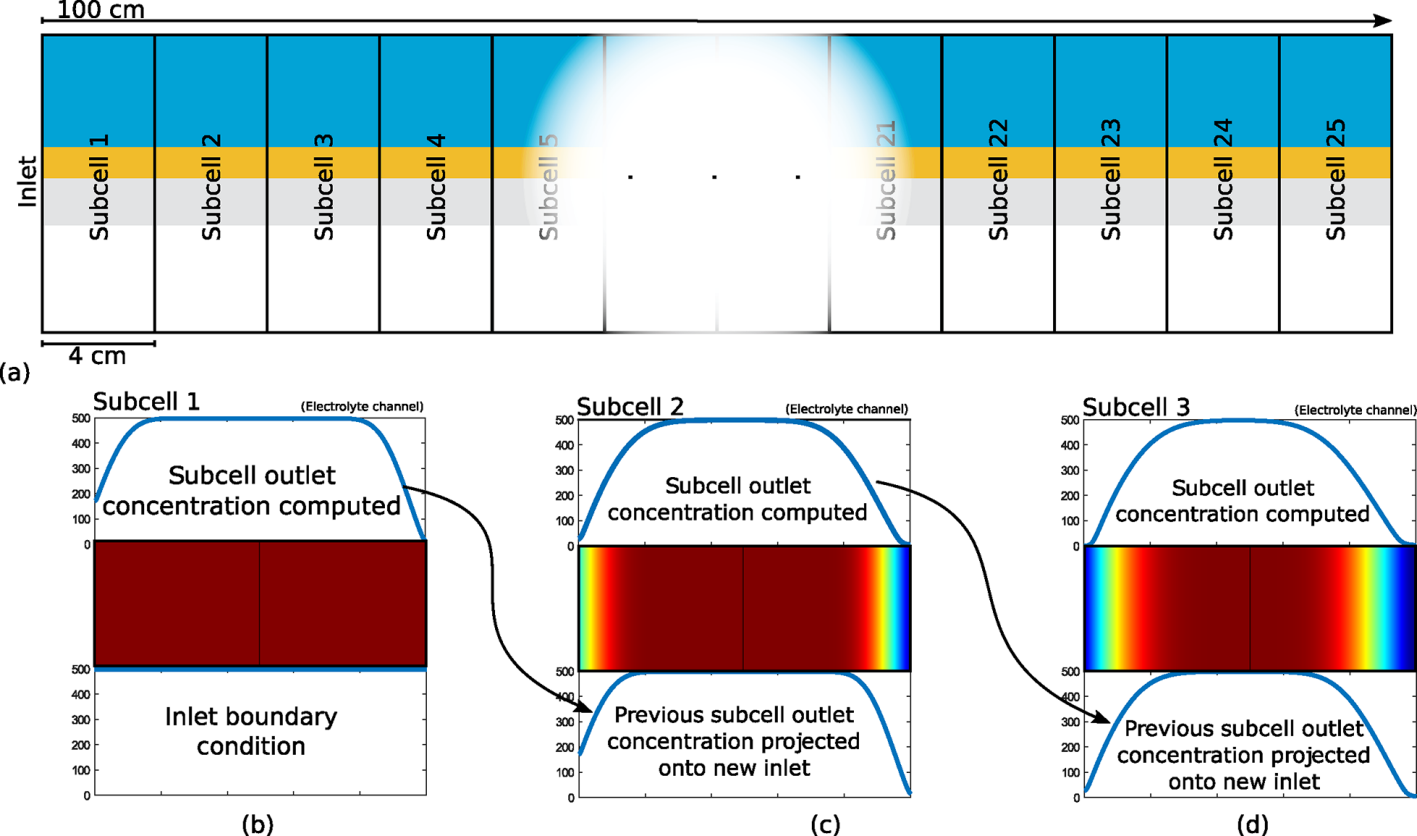
The simulation sequentially solves subcells divided in the flow direction. (a) Decomposition of the 100 cm cell into 25 adjacent 4 cm subcells. The initial and final subcells are coupled to inlet and outlet regions, respectively, and each internal subcell is coupled to the boundary values of the adjacent subcells. (b) The inlet HCO_3_^–^ profile is projected onto the inlet of the first subcell, solved along the length of the subcell, then projected from the outlet onto the inlet of (c), which is subsequently solved and projected onto the inlet of (d) and so on. The other concentration and potential profiles at the top of each subcell catholyte channel and CL are projected onto the inlets of each subsequent subcell. The same method is applied between subsequent GDL and gas flow channel inlets, projecting the mole fractions and pressures in the gas phase. The numerical conditions are shown in Figure 2.

Similarly we require that electrolyte potential varies little in the flow-wise direction. The characteristic length of a subcell far exceeds the thickness of the channel, so the flow-wise current density, *j*_*z*_, will be negligible with respect to the flow-perpendicular current density *j*_*x*_. The variation of electrolyte potential in the flow-wise direction depends on the variation of electrode reaction kinetics, and while this can be large due to high pH gradients, it is still small compared to the potential drop across the channel. While the subcells allow us to reduce the size of the computed domain in favour of iteration, the above requirements still motivate us to take as long a subcell as feasible, but the size of 4 cm is chosen out of expediency. While domain decomposition and variable transformations are not uncommon in numerical modelling they are underutilised in the field, with most models of electrochemical CO_2_ reduction resorting to 1D,^10,22,32,33^ simplifying the homogeneous reactions,^34^ investigating only low current densities,^35^ or only modelling small electrolysers.^23^Table 1 shows the geometric and operational parameters of the electrolyser, with unreferenced parameters measured from an in-house experimental setup.

**Table 1:** Geometric and Operational Parameters for the Base Model^a^

parameter	description	value	unit	ref
*p*_abs_	gas pressure	1	atm	
*T*	temperature	293.15	K	
*c*_KHCO_3__	catholyte concentration	500	mM	
Re	Reynolds number	200		
*L*	flow channel length	4	cm	
*W*_EL_	flow channel thickness	0.254	mm	
*W*_CL_	CL thickness	3.5	μm	(22)
*W*_GDL_	GDL thickness	325	μm	(36)
*W*_GAS_	gas channel thickness	3	mm	
*H*	flow channel height	5	mm	
ε_CL_	CL porosity	0.5		
ε_GDL_	GDL porosity	0.53		(36)
κ	GDL permeability	1.72 × 10^–11^	m^2^	(36)

a For a description of all parameters, see Table S2. Flow rate and channel length in the validation cases are adjusted accordingly, with standard temperature and pressure. Channel parameters are based on an in-house experimental setup with dimensions similar to that of Wu et al.^8^.

## Results

### Model Validation

**Figure 4:**
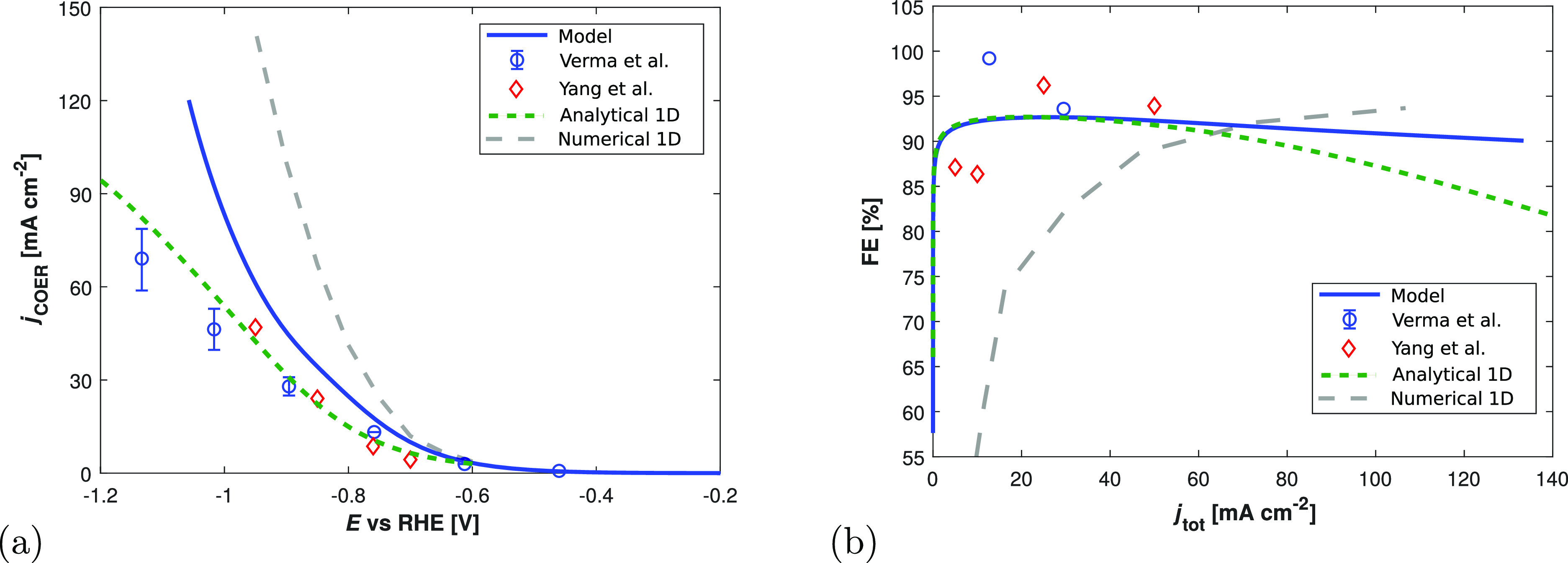
Model validation against experimental results from experimental results from Verma et al.^40^ as well as experimental results from a similar experimental setup from Yang et al.,^41^ and a computational model developed by Weng et al.^22^ and an analytical model developed by Blake et al.^33^ based on the electrolyser properties of the Verma et al. experiments. (a) Partial CO_2_ current density against cathode potential and (b) the Faradaic efficiency against total current density. The line plot for the computational model is for the experimental case of a 15 mm long flow electrolyser with a 0.5 M unsaturated KHCO_3_ liquid electrolyte with a liquid flow rate corresponding to Re = 2.6 and an excess gas feed.

**Figure 5:**
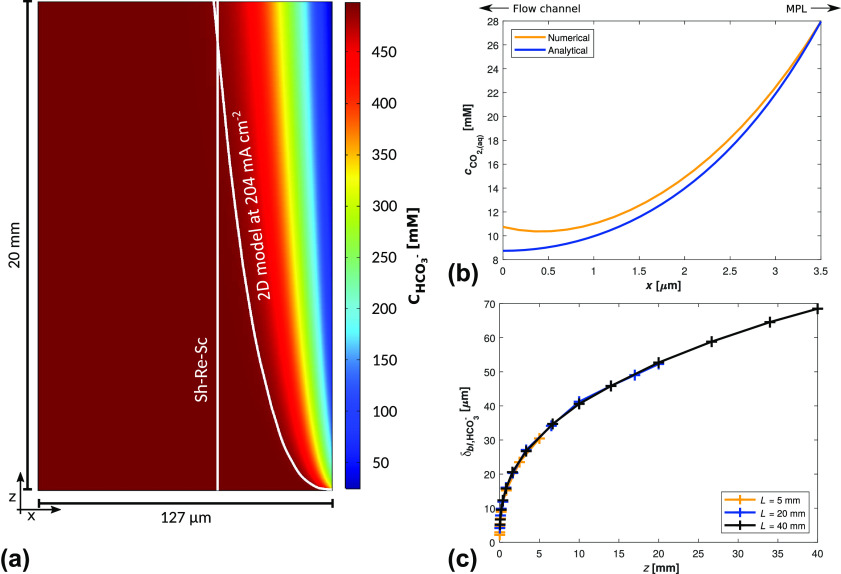
Analytical results for flow-wise development of concentration boundary layer thickness, compared to numerical results. To quantify the numerical boundary layer thickness, HCO _3_^–^ is used as the characteristic species to quantify the buffering effect, rather than CO_2_. In (a) the boundary layer develops from negligible thickness to a thickness greater than that of the Sherwood–Reynolds–Schmidt correlation (eq S2), which does support its validity as an average approximation, but also highlights how unreasonable it is to take such an average near the inlet. In (b) the CO_2_ distribution in the CL from the model follows  as is expected of a 1D reaction-diffusion system.^33,42^ More information on the analytical approximations can be found in SI, section 1.2. In (c) the boundary layer thicknesses exhibit a dependence on  as is expected of Poiseuille flow for boundary layers much thinner than the half-channel width.^43,44^

Figure 4 shows experimental comparisons of partial current density against cathode potential and FE against total current density, for the parameters in Table S2. The match between computational and experimental results is good, only slightly overestimating partial current density. It is expected that the deviation arises from phenomena outside of the scope of the model, such as significant bubble formation near the CL-electrolyte channel interface or liquid breakthrough in the MPL. Both of these phenomena would result in a reduction in cell performance due to reduced access to reaction sites^37^ and increased diffusion pathway lengths^38^ and are both present primarily at higher current densities. As the liquid breakthrough would lead to a significant reduction in FE,^39^ which is not observed in Figure 4b, the bubble formation explanation is favoured. Despite the short length of the validation case electrolyser, the difference between the presented 2D model and the 1D model of Weng et al. is stark. The use of one operation-independent boundary layer thickness in the Weng model leads to prohibitively low mass transfer between the electrolyte channel and CL at low current densities where the boundary layer should be thinner, and unrealistically high mass transfer at high current densities where the boundary layer should be thicker. By contrast the analytical model of Blake et al. predicts the development of the concentration boundary layer in the channel and takes an average value for its thickness, but neglects the effect that the buffer of the KHCO_3_ catholyte will have on this development. This leads to an overestimation of boundary layer thicknesses and a lower prediction for CO_2_ current density as a result. It is possible to verify against analytical mathematical approximations of scaling relations that can be extrapolated to larger length scales. Figure 5 shows that the computational results match the predicted trends well, such as the cubic root length scaling of the boundary layer thicknesses and the hyperbolic cosine shape of the CO_2_ distribution in the CL. The quality of the agreement with these results is the best validation that the circumstances permit.

## Upscaling

### Upscaling Study

To determine how local reaction environments change for longer electrolysers, we compare a 4 cm long electrolyser model with a 100 cm long electrolyser model. For this ratio of lengths, we chose to model the 100 cm electrolyser receiving a 25× higher CO_2_ flow rate in the gas channel due to it being 25× longer. Despite this, the pressure drop in the gas phase remains small compared to the liquid pressure drop. The liquid flow rate between models is held constant. Depending on the flow geometry, this can lead to large pressure drops in the liquid phase that far exceed the pressure drops in the gas phase, up to the order of hundreds of millibars for thin single channels. Although this could lead to many problems, from mechanical stability to local liquid breakthrough when pressure variations exceed the stability window of the GDL,^45^ these effects are outside of the scope of this work and are not modelled. The gas flow rate was varied to give insight into how different single pass conversions affect cell performance.

**Figure 6:**
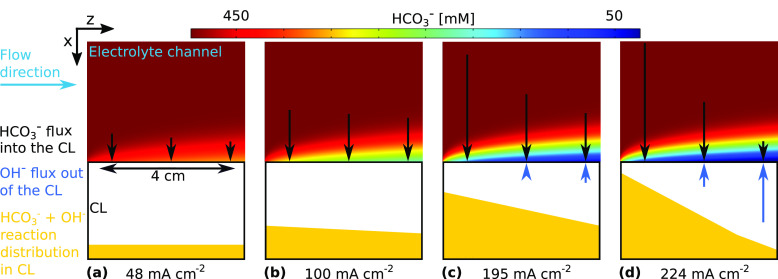
Profiles of HCO_3_^–^ just outside the CL at increasing total current densities, along with the respective distributions of the reaction in eq 5 of HCO_3_^–^ ions with OH^–^ in the CL. The yellow shape shows the distribution of the homogeneous reaction within the CL. In (a) and (b) relatively little carbonate buffering takes place due to low OH^–^ generation, and this reaction is quite uniform and almost exclusively takes place within the CL (>99%). For (c) and (d), however, the reaction rate is relatively high, occurs predominantly close to the inlet where CO_2_ availability and OH^–^ generation are highest, and increasingly takes place in the buffer layer outside of the CL, with 10% in (c) and 30% in (d). This increasing reaction outside of the CL is due to the combined transport of buffer into the CL and CO_2_ gas supply together being insufficient to buffer the generated OH^–^, which is now able to diffuse out into the electrolyte channel before reacting with the bulk buffer solution.

Given the focus on higher current densities (>100 mA cm^–2^), we normalise reactant consumption by gas phase reactant supply only, as the overwhelming majority of CO_2_ from the liquid flow channel is expected to be consumed in buffer reactions before reaching the CL for current densities above 40 mA cm^–2^.^46^ If the yield was normalised around the total influent CO_2_ from both gas and liquid phase then it would become a relatively low percentage that is dependent on the ratio of channel flow rates. This electrolyte contribution to the reaction is similar to the operational method of a bicarbonate reactor, but such reactors seldom reach high current densities and when they do, they require high electrolyte concentrations.^47^ We furthermore disambiguate between the usage of consumption and conversion following the convention of Larrázabal et al.,^48^ defining consumption as the percentage of gas phase CO_2_ that enters and is consumed in the electrolyser and conversion as the percentage of influent gas phase CO_2_ converted specifically into CO. These choices are further motivated by the results of the lab-scale electrolyser model in Figure 6.

### CO_2_ Conversion

At the low current density in Figure 6a, the carbonate buffer reaction in eq 5 occurs almost entirely within the CL and HCO_3_^–^ concentration remains high at the edge of the CL, indicating that the pH within the CL remains well buffered and the aqueous CO_2_ concentration is high enough for the reaction to be performed in both bicarbonate electrolyser mode and gas supplied mode. However, at higher current densities in Figure 6c,d, HCO_3_^–^ is depleted by the reaction in eq 5 before reaching the CL, and the buffering reaction takes place in a concentration boundary layer outside the CL, showing that the reaction can no longer be performed in bicarbonate electrolyser mode. Despite this, when tracking the total HCO_3_^–^ consumed this way we still find that the majority of the reaction takes place within the CL, implying that the buffering effect is actually due to the reaction of gas-supplied CO_2_ to HCO_3_^–^ through eq 4 and then CO_3_^2–^ through eq 5.

**Figure 7:**
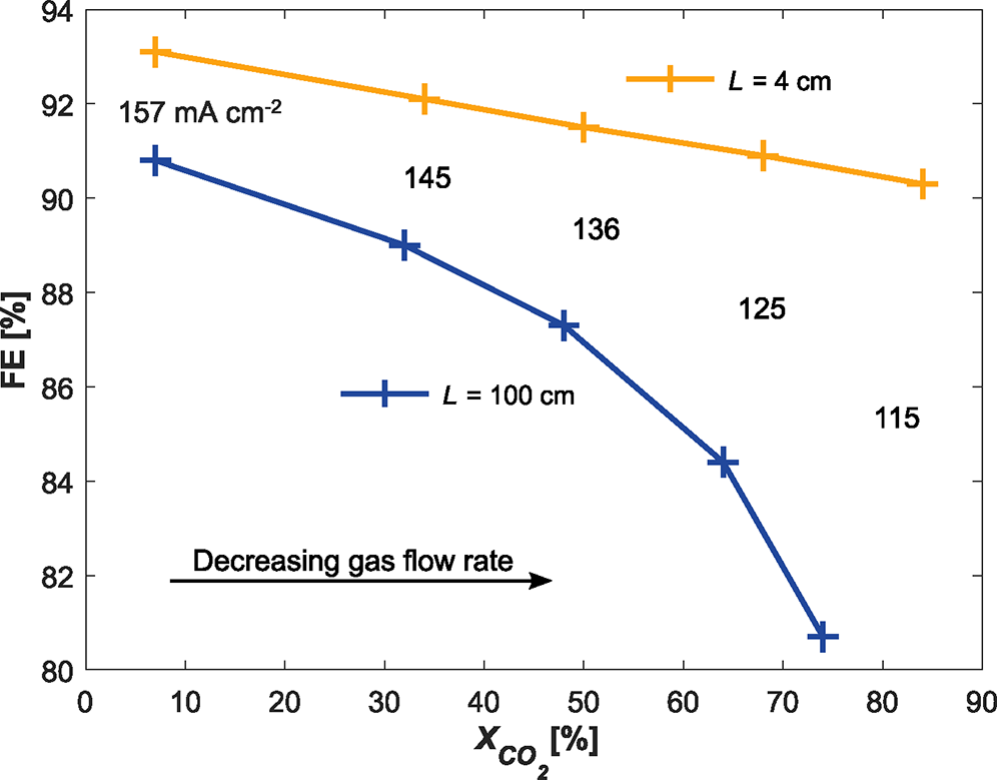
Effect of increasing single pass conversion,  on Faradaic efficiency in the 4 and 100 cm electrolysers. The 100 cm electrode is maintained at 3.1 V, leading to a progressive reduction in average *j*_tot_ as flow rate is reduced to emulate increasing single pass conversion. The 4 cm electrolyser cell potential is adjusted from 2.98 to 2.94 V to match the average *j*_tot_ at each corresponding gas flow rate. While FE decreases when single pass conversion is increased, the drop is more severe in the 100 cm case.

**Figure 8:**
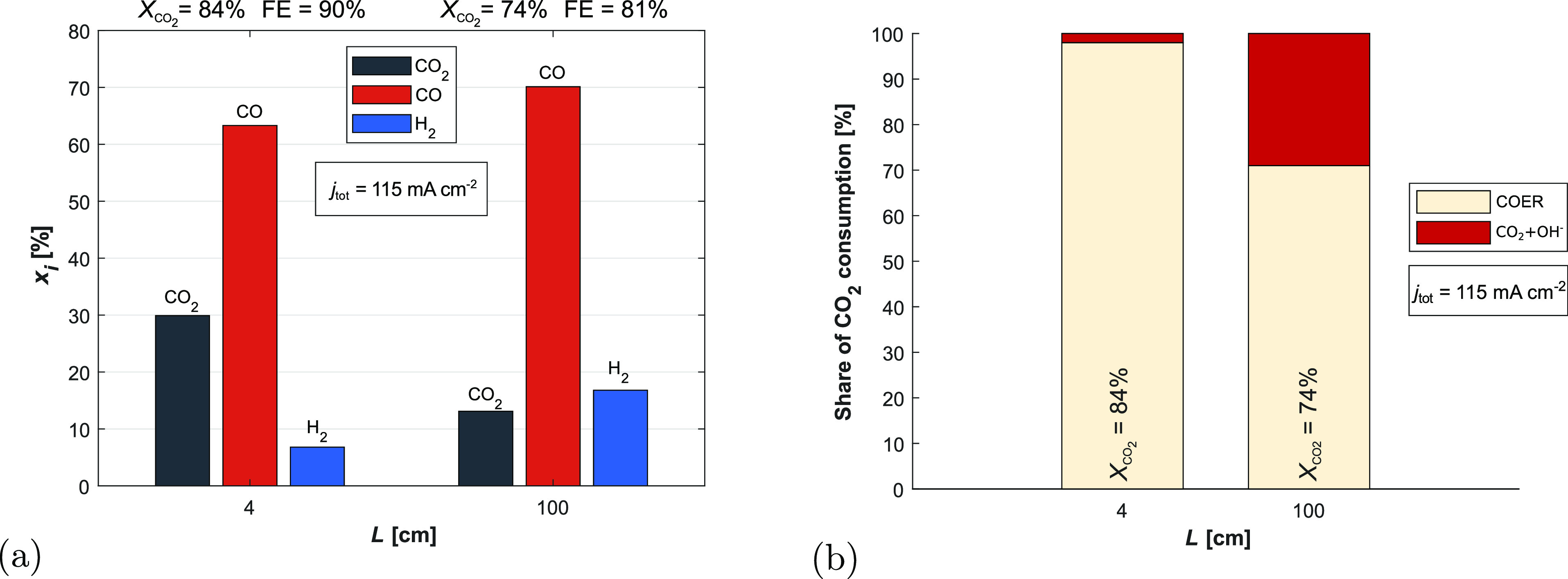
(a) Molar composition of the gas channel outlets in the two cases. Despite lower total CO_2_ conversion and higher H_2_ mole fraction, the 100 cm case exhibits a higher CO mole fraction in the gas outlet. (b) Relative magnitude of reaction sinks for CO_2_ entering the CL from the gas phase. This explains the behaviour in (a), as 30% of the total CO_2_ consumed is converted to HCO_3_^–^ through the reaction with OH^–^ instead of being converted to CO, leading to a higher total consumption of CO_2_ and a lower resultant CO_2_ fraction in the outlet stream. It is thus the loss of reactant rather than improvement in conversion that seemingly improves the outlet composition.

The performances at different single pass conversions are shown in Figure 7. At low conversions, the supply of CO_2_ is not limiting, and so both cases exhibit high FE, with the small reduction in the 100 cm electrolyser FE arising only from the increase in average boundary layer thickness, following the trend of the 4 cm case in Figure 5c. As conversion increases, the reduction in FE is far more severe in the 100 cm electrolyser than the 4 cm, as the reactant CO_2_ is increasingly consumed by the parasitic reaction with OH^–^ in the regions where the boundary layer is too thick to effectively buffer the OH^–^ produced in the CL. This can be seen directly in Figure 8b, in which 30% of the total consumed CO_2_ is converted into HCO_3_^–^ and CO_3_^2–^ instead of CO. This high consumption can be easily erroneously interpreted as a high conversion, as in Figure 8a, in which the outlet ratio of CO_2_ and CO seems much improved in the 100 cm case over the 4 cm case. It is instead the loss of reactant rather than an increase in product that skews this ratio.

Experimental studies often report a significantly higher consumption of reactant than is found in Figure 8b due to conversion to CO_3_^2–^, as well as crossover of this CO_3_^2–^ through the membrane. The reports of exceptionally high crossover mainly come from electrolysers with AEMs,^49^ but BPMs inhibit the transport of the carbon carrying anions,^14^ so we would expect lower crossover in our case. However, our assumption that BPMs entirely prevent this ion transport may not be realistic, as unwanted crossover has still been reported in BPM electrolysers.^50,51^ There is also an argument that purely by the stoichiometry of eqs 1, 4 and 5 and balancing the local production and consumption of CO_2_ and OH^–^, the minimum fraction of reactant CO_2_ converted to CO_3_^2–^ should be 50% .^48^ This argument assumes fast equilibrium reactions though, and Weng et al. showed that the CO_2_ utilisation efficiency is dependent on *k*_1_ from eq 4,^10^ and we also found that the kinetic treatment is necessary as eq 4 is out of equilibrium. The 50% minimum estimate is most valid for KOH electrolytes due to their high pH and capacity for CO_2_. We instead use a CO_2_ saturated KHCO_3_ electrolyte with a high buffer capacity, and although we do observe a large reaction between CO_2_ and OH^–^ a significant portion of this comes from the catholyte supplied CO_2_ while we only consider CO_2_ sourced from the gas phase in Figure 8b.

The impact of the chemical consumption of CO_2_ by the reaction with OH^–^ depends on the relative costs of the gas channel upstream and downstream processes. If the cost of separation of CO from the product stream is relatively high, then the excess CO_2_ consumption actually improves the economics due to the higher CO outlet portion in the 100 cm electrolyser. Assuming that the anolyte and catholyte streams are recombined and recirculated through the cell this chemically reacted CO_2_ will be converted back from CO_3_^2–^ and HCO_3_^–^ when the acidic anolyte stream shifts the pH back to the initial value and the CO_2_ can be recovered from the solution. This means that the reaction with OH^–^ need not be considered as a true loss of reactant, rather as an inhibiting process that reduces *j*_CO_ and single pass conversion.

### Reaction Inhomogeneity

**Figure 9:**
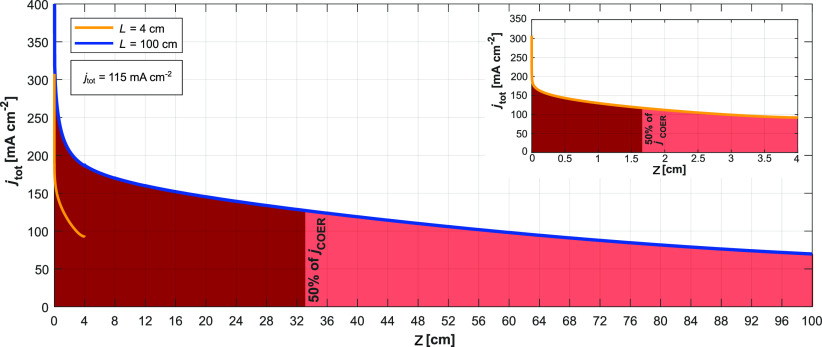
Local values of *j*_tot_ in the two electrolyser cases for an average total current density of *j*_tot_ = 115 mA cm^–2^. The 100 cm reaches a higher value of *j*_tot_ near the inlet due to the higher cell potential (3.1 V) than the 4 cm case (2.94 V) necessary for the same average *j*_tot_, but falls to a lower value near the outlet due to the larger pH increase in the CL.

The variation of *j*_tot_ is also greater in the 100 cm case. Figure 9 shows *j*_tot_ near the inlet, in the vicinity of which the boundary layers are thin and the CL is very well buffered. At the inlet, *j*_tot_ is far greater than *j* at the outlet, where both poorer buffering and reduced reactant supply. This ideal inlet region is limited only by overpotential and as such reaches a higher value of *j*_tot_ in the 100 cm case due to the higher cell potential necessary to reach an average of 115 mA cm^–2^. Despite this increased potential, *j*_tot_ at the outlet drops below the 4 cm case, because the pH is far higher in the 100 cm case. This inhomogeneity of the reaction distribution leads to the half of the total CO_2_ reduction reaction taking place in only the first third of the electrolyser. Additional discussion of the pH gradients in the CL and catholyte channel can be found in SI, Section 2.

The inhomogeneity of the reaction distribution and failure of the buffering effect also causes a wide range of pH values to be present along the CL. The well-buffered inlet will retain a pH equal to that of the influent electrolyte, but the poorly buffered CL near the outlet will have a far higher pH unless the current density is very low or the gas phase CO_2_ supply is very high. This increase in pH leads to a larger equilibrium potential correction through eq 15, a greater parasitic reaction rate and a chemical shift towards CO_3_^2–^. The electroneutrality condition requires that the high CO_3_^2–^ concentration be paired with a high K^+^ concentration, which together facilitate the precipitation of K_2_CO_3_ on the solid phase, leading to a reduction in diffusion pathways for CO_2_ and potential damage to the porous structure as the K_2_CO_3_ crystals obstruct pores in the CL and MPL.^39,52^ These precipitates are also hydrophilic and can, along with the high pH, alter the potential of zero charge and thus electrowetting properties of the solid–liquid boundary, leading to loss of effective hydrophobicity in the MPL. This allows electrolyte breakthrough into the GDL and gas channel, leading to a negative impact on FE and stability. The penetrating electrolyte increases the diffusion pathway length for CO_2_ as it blocks pores, leading to a reduction in *j*_CO_ while allowing HER to continue unmitigated. This pervasive issue is one of the main causes of performance loss over time, and mitigation strategies currently focus on temporal variation in reactor operation or low electrolyte strengths.^53,54^

### Discussion

By modelling the flow parallel direction as well as the through plane direction, we overcome the limitations of 1D models that require empirical or simplified analytical expressions to attempt to capture flow-wise variation. The work of Yang et al.^55^ includes the flow channel in 2D but neglects variation in pH in the electrolyte. This is at odds with this work and the work of Kas et al.,^23^ which both predict large variations in local pH. While this could be roughly justified by the use of high pH KOH electrolyte, it would still have a large impact on the local homogeneous reaction rates and the total loss of reactant to carbonate formation. This is not reported on, despite carbonate crossover being a serious issue,^14,49−51^ and despite the homogeneous reaction kinetics they report being significantly faster than the heterogeneous kinetics for such a high pH. This assumption of pH homogeneity also hampers the upscaling analysis, as homogeneous properties are indifferent to scale, giving overambitious positive predictions for scalability. By contrast, the experimental scalability analysis of Jeanty et al.^56^ highlights the more fundamental issues of electrolyte breakthrough and salt precipitation. Both of these support our conclusion of the importance of quantifying local pH and its effect on the carbonate equilibrium reactions as the perspired electrolyte has a pH value of 10 compared to that of the neutral K_2_SO_4_ catholyte and the precipitate comprised of carbonate salts for which the carbon could only have been sourced from the CO_2_ feed. They also find similar trends for FE, in which the upscaled electrolyser suffered a larger reduction in FE when lowering feed gas CO_2_ supply. The most interesting result is the drastic improvement when implementing the circulating pump in the scaled up cell which, despite lowering the average CO_2_ concentration, leads to a much more uniform concentration profile and a break up of the boundary layer. We identify an analogous issue in the catholyte channel with nonuniform pH and thick boundary layer development as a primary concern in scalability, and the experimental success in reducing the effect in the gas channel is reassuring.

#### Stability

The extension of the 4 cm model to the 100 cm model shows deterioration in conversion, FE, and necessary cell potential, and the model indicates that these issues will only be exacerbated with further elongation of the flow channel. While these performance losses could be in some cases surmountable, the extreme variation in local reaction environment that comes with them would lead to further issues outside of the scope of the model. While liquid breakthrough in generalised porous media is well understood, electrolyte breakthrough in the electrolytic cell is related to precipitation and electrowetting. Precipitation would require stochastic modelling and electrowetting effects are poorly understood at interfaces where charge transfer reactions take place, so both are out of the scope of the model. However, we can note that both are dependent on the local ionic concentrations and pH, the latter due to the shift in potential of zero charge, and comment that by determining the general trends in pH and ionic strength we can predict that the upscaled model will be more susceptible to breakthrough closer to the outlet. In the future, when better descriptions of these phenomena are available, it will be possible to bridge the gap between local environment scalability and pressure stability studies^45,57^ to create a full description of breakthrough.

If the Reynolds number of the electrolyte flow channel is held constant, then the pressure drop along the channel will increase linearly with electrolyser length. The pressure variation in the liquid phase can lead to electrolyte or gas breakthrough when out of balance with the gas pressure drop, so it is ideal to scale one flow rate to ensure the differential pressures remain within MPL stability ranges. If particularly thin flow channels, such as those used in lab scale electrolysers, are required to minimise ohmic losses then the pressure drop can become excessively large. Without sufficiently pressure resistant MPLs this could lead to liquid breakthrough. Both the hydrophobicity and the mechanical stability of CLs are dependent on ionomer type and loading, which vary between experimental studies and are the subject of independent optimization. Furthermore, it becomes an issue to mechanically support a flow channel under such conditions, potentially necessitating additional structural supports in the flow channel, which could cause the Poiseuille flow to transition into plug flow. Plug flow boundary layers scale with , rather than the  of Poiseuille flow,^58^ and as such can grow more rapidly. Specifically, we can take the ratio of the Lévêque approximation for plug flow and Poiseuille flow concentration boundary layer thicknesses,
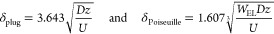
32where the concentration at the edge of the boundary layer is 99% of the bulk concentration. Plug flow will lead to thicker boundary layers when
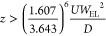
33which in our case for the diffusivity of HCO_3_^–^ is roughly after only 11 cm. Widening the channel leads to increasing ohmic drop across the electrolyte, so a balance must be struck between electrolyte flow rate, pressure drop, and ohmic drop. For small lab scale electrolysers this balance usually favours thin channels due to the short flow channels exhibiting small pressure drops and little variation in the local pH and boundary layer, but as electrolyser length increases the benefit of lower ohmic drop will be outweighed by the cost of reduced GDL stability and increase in boundary layer thickness. This trade-off between flow velocity, flow channel length, and ohmic drop is an optimisation problem that will remain relevant even in a full-scale stack implementing short channels.

#### Performance Loss Minimisation

With the sources of the performance losses isolated, it is possible to propose potential solutions. The primary issue is the lack of effective buffering due to thick concentration boundary layers, and so reducing the boundary layer thickness is paramount. This could be done by introducing static mixers or altering the geometry in the electrolyte channel to induce chaotic mixing^59^ and break up the developing boundary layer, and although this could worsen carbonate and product crossover,^60,61^ product crossover is only likely to be an issue when producing primarily liquid products.^62,63^ A similar issue would arise from the use of a stronger base such as KOH, in which the high pH would lead to unavoidable loss of CO_2_ feed reactant to carbonate. When averaging across the entire CL for 115 mA cm^–2^, we find a dissolved CO_2_ concentration of ∼5 mol m^–3^, so as a rough estimate, we can replace our predicted pH of ∼12.5 with 14 for KOH to find from eqs 9 and 19 that the percentage of total reacted CO_2_ reacting with OH^–^ rises from ∼30%, in Figure 8b, to ∼80%. This chemical reaction with OH^–^ would be significantly greater than the electrochemical production of OH^–^, leading to a decreased pH boundary layer forming in the flow, in which the gas CO_2_ feed is used primarily to buffer the KOH. This boundary layer would be comprised of carbonate and bicarbonate and suffer from lower conductivity, and in a recirculated electrolyte would eventually react with enough feed CO_2_ to effectively become KHCO_3_. In this case the share of CO_2_ converted to CO would actually be improved by increasing overpotential at the cost of FE.

Circumvention of scaling issues can be preferable to prevention, so alternative parallelised geometries can be employed to retain short channels and high performance. Splitting one long channel into multiple shorter channels would require each shorter channel to maintain a comparable flow rate to and thus pressure drop to achieve greater performance, so it would be preferable to use entirely distinct shorter channels with separate inlets and outlets. While techno-economic assessments often consider MEAs without catholyte buffers, Badgett et al. showed that the inclusion of a catholyte buffer layer improves performance^64^ and only requires an increase in the capital cost of the system by around 1%.^65^ The supply of catholyte at sufficient flow rates to multiple channels within a cell or stack could necessitate additional equipment and, from a holistic perspective, could complicate full-scale stack designs.

Alternatively, the problem can be addressed at the source: the high production rate of OH^–^ ions. While the production of OH^–^ due to the reduction reaction (eq 1) is necessary and unavoidable, the low FE shows that the HER (eq 2) rate is unnecessarily high, especially in the regions with low *j*_CO_. The low *j*_CO_ is due to reactant limitation, not lack of available reaction sites, so by reducing the catalyst loading further along the electrolyser the zeroth order HER would be reduced linearly but the COER would be impacted less as it would remain reactant limited. This would cause an overall reduction in single pass conversion, but an improvement in FE and reduction in parasitic consumption as the lowered OH^–^ production from HER would lower the pH in the CL. Variable catalyst loading the flow direction will be discussed in more depth in a forthcoming paper, and a brief demonstration of the efficacy of the proposed solution can be found in SI, Section 3.

## Conclusion

We have developed a large-scale 2D computational model of a CO_2_ reduction flow cell, and showed through comparison of a 4 cm lab scale model and a 100 cm upscaled model that the performance metrics of FE, conversion, and voltage efficiency decrease with electrolyser length. We found that poor electrolyte buffering due to increasing boundary layer thickness in the flow channel leads to reaction inhomogeneity in the flow direction and poor utilisation of the outlet regions of the electrolyser, resulting in a system that struggles to match performance benchmarks set in lab scale electrolysers. While some mitigation strategies have been suggested, such as electrolyte mixing, catalyst loading variation, and flow channel optimisation, it is recommended that future experimental studies test these strategies and acknowledge that not only will the necessary current densities for industrial realisation be greater than those commonly assumed in lab scale studies, but the local reaction environments will be far less favourable than those present in typical lab scale electrolysers.

## Supporting Information Available

Description of analytical methods, additional parameters, and additional plots of local environment and variable catalyst loading (PDF)
