# Supplementary material for: Inhomogeneities in the Catholyte Channel Limit the Upscaling of CO2 Flow Electrolysers
Source: ACS Sustain Chem Eng. 2023 Feb 7;11(7):2840–52. doi: 10.1021/acssuschemeng.2c06129 (PMC9945194; doi:10.1021/acssuschemeng.2c06129)
Supplement: Supplementary file 1 — sc2c06129_si_001.pdf [file sc2c06129_si_001.pdf]

Supporting Information: Inhomogeneities in the catholyte channel limit the upscaling of CO_2 flow electrolysers

Joseph W. Blake*†, Vojtěch Konderla‡, Lorenz M. Baumgartner‡, David A. Vermaas‡, Johan T. Padding†, J. W. Haverkort†

†*Department of Process and Energy, Delft University of Technology, Leeghwaterstraat 39, 2628 CB Delft, The Netherlands*

‡*Department of Chemical Engineering, Delft University of Technology, 2629 HZ Delft, Netherlands*

– Number of pages: 10

– Number of figures: 7

– Number of tables: 2

Contents

1 Additional Theory **S1**

1.1 Central channel continuity S1

1.2 Analytical comparison S2

1.3 Salting-out S2

2 Additional Plots **S3**

3 Variable catalyst loading **S7**

4 Parameters **S9**

5 Numerical methods **S10**

1 Additional Theory

1.1 Central channel continuity

As the electrolyte flow channel is decomposed into two subchannels, the boundaries must be specified. It is assumed that the boundary layer thicknesses at each side of the channel are sufficiently less than half the channel width so that the electrolyte is fully equilibrated at the centre and the electrolytic composition in the neighbourhoods of the mid-channel divide are equal. In the case where boundary layer thicknesses are comparable to the half-channel width, the computational domains can be slightly reshaped as the boundary layer development at the membrane will differ from that at the catalyst layer boundary due to it being dependent on different species with different diffusivities: CO_2 and H^+ at the membrane and CO_3^{2-} and OH^- at the catalyst layer. Continuity at the mid-channel divide is simply given by

$$c_{i,\text{left}} = c_{i,\text{right}}, \tag{S1}$$

and as the acidic and alkaline homogeneous reaction pathways share equilibrium constants this should be trivially true provided the boundary layers do not approach the divide.

*Corresponding author: J.W.Blake@tudelft.nl

1.2 Analytical comparison

In Fig. 5(a) in the main text, the Reynolds-Sherwood-Schmidt correlation [1] used for the mass transfer boundary by Weng et al. in Ref. [2] is given by

$$k_{\mathrm{MT}} = 0.664\frac{D}{L}\left(\frac{\rho_l U L}{\mu_l}\right)^{\frac{1}{2}}\left(\frac{\mu_l}{\rho_l D}\right)^{\frac{1}{3}}, \tag{S2}$$

and is a similarity solution, original derived for heat transfer, in co-developing momentum and diffusion boundary layers. It is questionably relevant for the flows we consider, as the hydrodynamic entrance length is small and often comparable to the flow inlet region, so the momentum boundary layer is likely only co-developing for a small portion of the channel length. The computational model however, follows a curve closely matching the Lévêque approximation [3, 4], which is derived from the advection-diffusion equation neglecting co-flow diffusion, and is given by

$$\delta = 1.607\left(\frac{W_{\mathrm{EL}} D z}{U}\right)^{\frac{1}{3}} \tag{S3}$$

for Poiseuille flow. This roughly leads to the $z^{\frac{1}{3}}$ dependence seen in Fig. 5(a) and Fig. 5(c) in the main text. However, the advection-diffusion equation does not include the effect of homogeneous reactions. A more detailed description of this can be found in Lin et al. [5] in which a bicarbonate buffer system is included through a modified Graetz-Lévêque approach leading to a modified Lévêque scaling for Sherwood number multiplied by a stoichiometry dependent reaction factor ($\frac{1}{2}$ in the diffusion limited regime) in parallel with the surface reaction Damköhler number. For diffusion limited regimes, this means that $z^{\frac{1}{3}}$ scaling should be retained, but we do not attempt to include more details due to the differences in geometry, with the analytical model more closely representing an H-cell than a gas-diffusion electrode, and the incomplete treatment of the buffer ion transport. This work does not include electromigration however. The analytical approximation in Fig. 5(b) in the main text comes from the solution to the 1D reaction-diffusion equation laid out in Blake et al. [6],

$$-D\frac{\partial^2 c}{\partial x^2} = \frac{a_v i}{2F}\frac{c}{c_{\mathrm{ref}}}\exp\left(\frac{-\eta}{b}\right) + \epsilon k_1 c \langle c_{\mathrm{OH^-}}\rangle, \tag{S4}$$

in which only the fast homogeneous forward reaction between OH^- and CO_2 and the heterogeneous reaction are considered. Collapsing the reaction rate terms into a single first order reaction rate constant k, the solution can be written as

$$c = c_0 \frac{\cosh \mathrm{M_T}(1 - \frac{x}{W_{\mathrm{CL}}})}{\cosh \mathrm{M_T}} \tag{S5}$$

where $\mathrm{M}_T = \sqrt{\frac{k W_{\mathrm{CL}}^2}{D_{\mathrm{CO_2}}}}$ is known as the Thiele modulus and

$$k = \frac{a_v i}{2F}\frac{1}{c_{\mathrm{ref}}}\exp\left(\frac{-\eta}{b}\right) + \epsilon k_1 \langle c_{\mathrm{OH^-}}\rangle \tag{S6}$$

. While in the original work, the OH^- concentration is simultaneously determined analytically, in this work we simply insert the computationally determined average OH^- concentration to yield the analytical CO_2 profile in the CL.

1.3 Salting-out

The Sechenov equation effectively modifies Henry's constant, $\mathcal{H}_{\mathrm{CO_2},0}$ in the presence of high ion concentrations, and can be written as

$$\mathcal{H}_{\mathrm{CO_2}} = 10^{-\left(S_{\mathrm{KHCO_3}} c_{\mathrm{HCO_3}^-} + S_{\mathrm{K_2CO_3}} c_{\mathrm{CO_3}^{2-}} + S_{\mathrm{KOH}} c_{\mathrm{OH^-}}\right)} \mathcal{H}_{\mathrm{CO_2},0} \tag{S7}$$

where the values of S_i can be determined from measurements and methods detailed by Schumpe et al. [7]. This formulation builds on the one used in [6], correcting a misuse of the natural logarithm, but attaining similar trends for ionic strengths at which the equation is valid.

**Salt**	S [$m^3\,kmol^{-1}$]
$KHCO_3$	0.1579
K_2CO_3	0.2802
KOH	0.1451

Table S1: Values of S for the three salts in the electrolyte

2 Additional Plots

The pH within the CL develops exceptionally quickly in the 100 cm case due to the increased potential, as seen in Fig. S1, leading to a pH gradient in excess of the entire 4 cm case within the first subcell. While the pH stabilises further along the electrolyser, this has to be qualified with the knowledge that the CO_2 current density is rapidly decreasing over the same length. This reduction in current density should lead to a reduction in OH^- generation and pH, but the depletion of the buffer and thickening of the concentration boundary layer prohibit this, as seen in Fig. S2. In the 100 cm case the pH increase is so severe that the variation in pH passes through the half channel line. It is urgent to note that, while the channel is divided into two sections for the purposes of modelling, this does not mean that the pH profile stops developing past this point. The diffusion, migration, and homogeneous reactions of species still occur, but the pathway for homogeneous kinetic reaction rate is determined by an acidic pathway (i.e. reaction with H^+) rather than alkaline. This only has an effect on the results when the reaction of CO_2 to HCO_3^- is out of equilibrium, which only occurs in the CL or at the BPM. At the half channel mark, the pH is sufficiently close to neutral that homogeneous reactions remain in equilibrium, and so the trespass of the pH boundary layer over the channel midpoint is not expected to cause any error.

The concentration overpotential can be written by collapsing the reactant term into the exponent and normalising by the Tafel slope, b, to arrive at

$$i_{\mathrm{COER}} = i_{0,\mathrm{COER}} \exp\left\{ \frac{b \ln \frac{c}{c_0} - \eta}{b} \right\}, \tag{S8}$$

where η is the activation overpotential and c and c_0 are the local and equilibrium concentrations of CO_2, not to be confused with the reference concentration of 1 M used in the Tafel equation concentration dependence. This is simply a consequence of the way the kinetics were derived, and the solution is to re-scale the exchange current density by a the ratio of the reference and equilibrium concentrations. The equilibrium concentration in this case in this case is set to the equilbrium value given by the Henry constant above (roughly 34 mM). We can further separate out these terms into the Nernstian shift in overpotential at the bulk electrolyte pH,

$$V_{\mathrm{N},0} = E_{0,\mathrm{COER}} - \frac{\ln(10) RT}{F} \mathrm{pH}_{\mathrm{bulk}} \tag{S9}$$

the Nernstian shift in overpotential at the local electrolyte pH

$$V_{\mathrm{N}} = E_{0,\mathrm{COER}} - \frac{\ln(10) RT}{F} \mathrm{pH}, \tag{S10}$$

and the effect of reactant depletion, normalised as a potential,

$$V_{\mathrm{CO_2}} = b \ln \frac{c}{c_0}. \tag{S11}$$

The negatives of these are plotted in Fig. S3 and Fig. S4, both for the region of the CL closest to the electrolyte channel (x = 0) and for the region of the CL closest to the MPL (x = W_{CL} We find that for high conversions the largest variation in overpotential is invariably from V_{CO_2} as reactant is depleted along the electrolyser. However, for the 100 cm case, the change in pH within the first few centimetres is so large that it exceeds the effects of concentration overpotential for the majority of the length of the electrolyser.

In the 100 cm case it can be seen that the pH and Nernstian potential both decrease slightly towards the outlet. Despite the widening boundary layer, the decrease in available gas-supplied CO_2

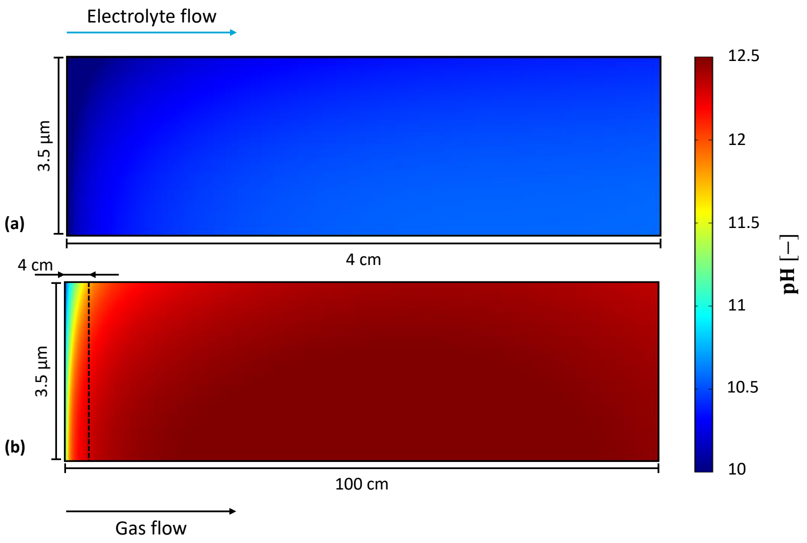

Figure S1: The pH within the CL for (a), the 4 cm case, and (b), the 100 cm case. The local j_{tot} in the first 4 cm of the 100 cm model far far exceeds that of the 4 cm model, and as such the pH increase is far greater. The combined effects of the Nernstian potential shift and increased reaction with OH^- lead to a lower j_{tot} across the rest of the of the electrolyser.

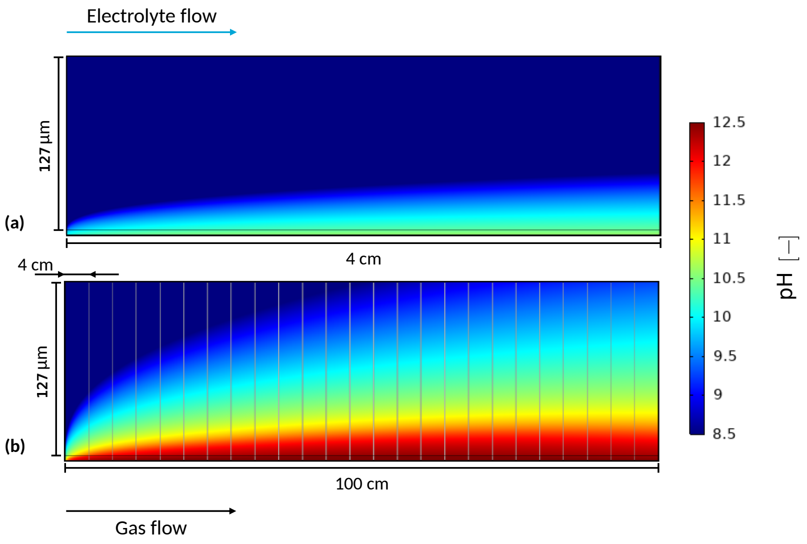

Figure S2: The pH within the cathode half of the catholyte channel for (a), the 4 cm case, and (b), the 100 cm case, with the subcells indicated in the 100 cm case. The 4 cm model only reaches a low pH and is short enough that the boundary layer only spans a small portion of the channel thickness. However, the 100 cm model pH boundary layer again grows far more rapidly even in the first 4 cm subcell, and continues to do so until the pH change begins to reach the half-channel divide.

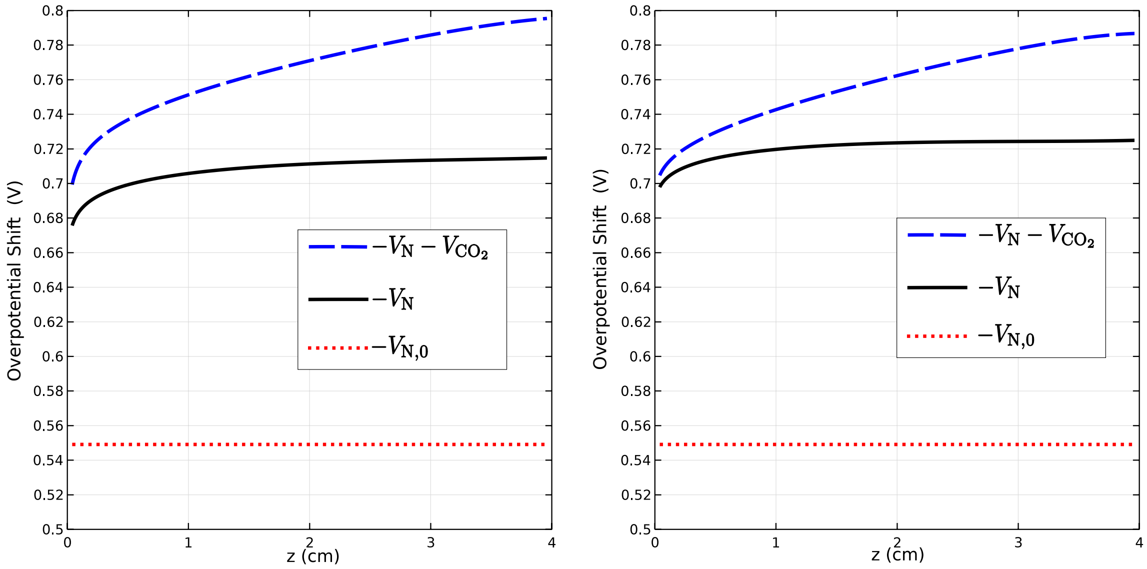

(a) Overpotential contributions at x = 0 in the 4 cm electrolyser model.

(b) Overpotential contributions at the at x = W_{CL} in the 4 cm electrolyser model.

Figure S3: Comparisons of overpotential contributions and development along the 4 cm long electrolyser model at 115 mA cm^{-2} and χ_{CO_2} = 84% at (a), the channel-CL boundary, and (b), the CL-MPL boundary. The contribution of V_{CO_2} to the total overpotential is greater at the channel than at the MPL boundary due to depletion through the CL. In both cases however, the increase in V_{CO_2} exceeds the increase in V_{Nernst} before at most 1 cm of the electrolyser length, showing that performance losses are almost always dominated by reactant supply at high conversions.

at high conversion causes a decrease in the amount of OH^- produced in the CO_2 electroreduction reaction. As we find a figure of only 30% non-electrochemical consumption of the CO_2 gas stream, this means that a reduction in available CO_2 leads to a net reduction in OH^- production. For example, a simplified scenario neglecting buffering catholytes and mass transport would see two CO_2 molecules entering the CL, one being reduced to CO and producing two OH^-, while the other reacting with two OH^- to form HCO_3^- then CO_3^{2-}. In this case the increase or reduction of CO_2 supply has no effect on pH. However, in our case only 30% of the gas-supplied CO_2 reacts with OH^-, so the same two CO_2 molecules would instead lead to one 1.4 CO ions and 0.6 CO_3^{2-} ions, leaving behind 1.6 OH^- ions. This possible as, despite the large concentration boundary layer, the excess OH^- is still buffered away by the HCO_3^- diffusing towards the CL.

The local Faradaic efficiency is shown in Fig. S5. While the drop in FE for the 4 cm case is almost entirely due to gas phase reactant depletion, the drop in the 100 cm case is also due to the significant parasitic consumption of reactant through the homogeneous reaction with OH^- at the higher local pH. While this parasitic reaction is still small compared to that observed in high pH catholytes or unbuffered systems, it is compounded by the higher potential necessary to reach the same current density, allowing higher rates of hydrogen evolution to continue unabated in the poorly utilised CL near the outlet. While the potential necessary to achieve the same average current density is higher in the 100 cm case, this only allows it to exceed the current density and FE of the 4 cm case directly at the inlet, z = 0. The resulting change in pH causes some odd behaviour in the FE profile, as we observe a Nernstian shift and a fast transition from a bicarbonate reactor operation mode to a gas-diffusion operation mode. In the 100 cm case this is all resolved within the first 4 cm subcell so subsequent subcells exhibit FEs that develop in a smoother manner, but in the 4 cm case the the lower current density at the inlet means that the pH gradients are small compared to the effect of reactant depletion and the FE develops smoothly throughout. The reactant depletion necessary for high conversion is why the FE after 4 cm is lower in the 4 cm case than the 100 cm, as the latter case has a correspondingly upscaled gas flow rate: a fairer comparison is shown in the insert in Fig. S5.

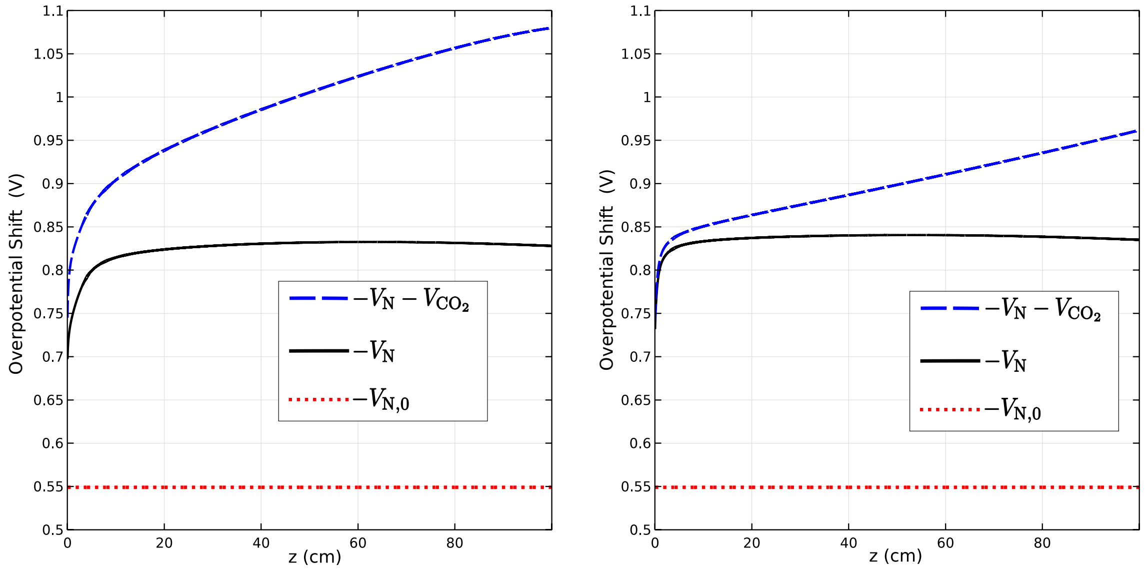

(a) Overpotential contributions at x = 0 in the 100 cm electrolyser model.

(b) Overpotential contributions at x = W_{CL} in the 100 cm electrolyser model.

Figure S4: Comparisons of overpotential contributions and development along the 100 cm long electrolyser model at 115 mA cm^{-2} and χ_{CO_2} = 74% at (a), the channel-CL boundary, and (b), the CL-MPL boundary. Similarly to Fig. S3, the profile for shows V_{CO_2} quickly dominates near x = 0. However, the variation in pH over the first few centimetres is so high that the contribution to V_{Nernst} exceeds that of V_{CO_2} for over half of the electrolyser.

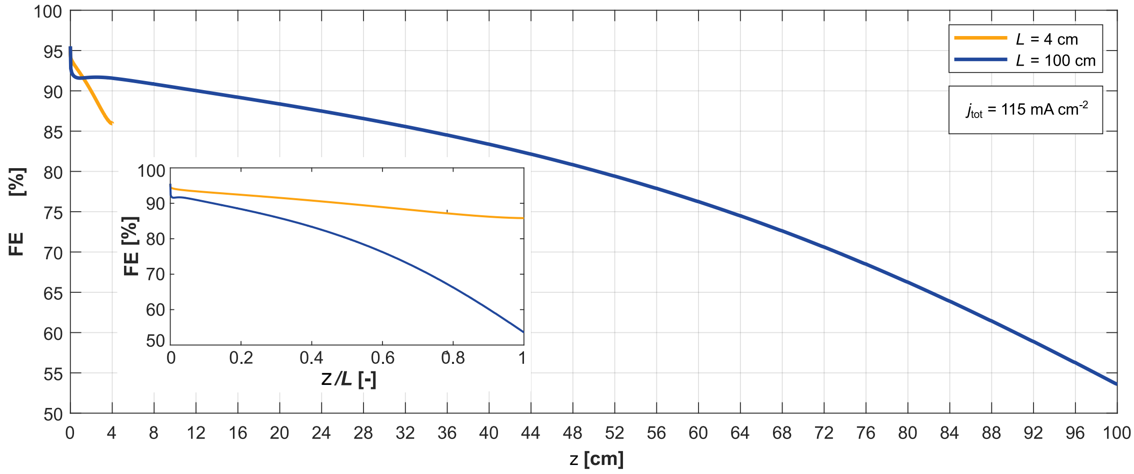

Figure S5: FE as function of the electrolyser length, FE(z), at 115 mA cm^{-2} for the 4 cm and 100 cm cases, with an embedded length normalised plot. While the 4 cm case exhibits a rapid drop in FE, this is due to the high conversion (χ_{CO_2} = 84%) and consequent depletion of reactant. When normalised by channel length we see that the drop in FE for the 100 cm case is far more dramatic, despite lower conversion (χ_{CO_2} = 74%).

3 Variable catalyst loading

One of the proposed methods of performance loss mitigation is to vary the catalyst loading in the flow direction. While the desired effect is to improve FE by reducing the amount of catalyst used predominantly for hydrogen evolution in the reactant limited regions in the latter half of the electrolyser, this has the secondary effect of reducing the pH through decreasing the OH^- production in both HER and COER. This reduction in pH reduces the Nernst potential shift and reduces homogeneous reaction rates, leading to a sublinear drop in COER rate with respect to catalyst loading. A schematic of the demonstrative simulation can be found in Fig. S6, in which

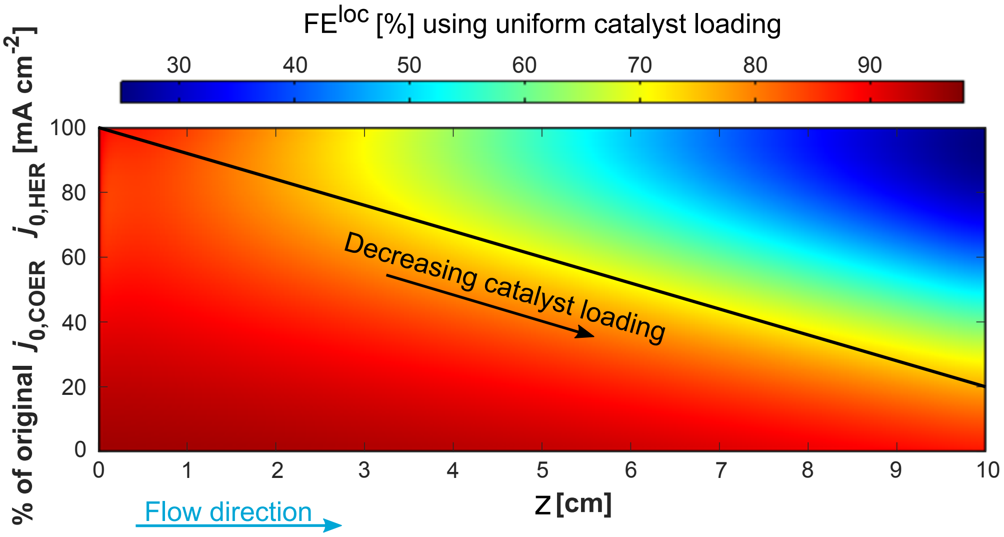

Figure S6: In a 10 cm the Faradaic efficiency is highest near the MPL-CL boundary and near the well-buffered inlet region of the electrolyser. Near the outlet and the flow-channel boundary however the high pH and low reactant concentration cause a drop in Faradaic efficiency to below 30%. To circumvent this issue we propose linearly decreasing catalyst loading down to 20% of the initial loading to reduce unnecessary HER and OH^- production in the poorly performing regions.

catalyst loading is linearly varied from unity at the inlet to 20% of inlet value in a 10 cm cell. This is achieved by linearly decreasing the exchange current densities of COER and HER, $i_{0,\text{COER}}$ and $i_{0,\text{HER}}$ respectively, linearly down to 20% of the value listed in the parameter table. In Fig. S7 the results show the improvement that even a simple unoptimised implementation of variable catalyst loading can achieve. At relevant current densities, where the reaction becomes reactant limited, the reduced loading case exceeds the CO_2ER current density at a higher FE, and with only 60% of the catalyst used in the control case. This improvement is expected to become even more pronounced in longer cells with lower flow rates, in which reaction inhomogeneity and low FE are more severe.

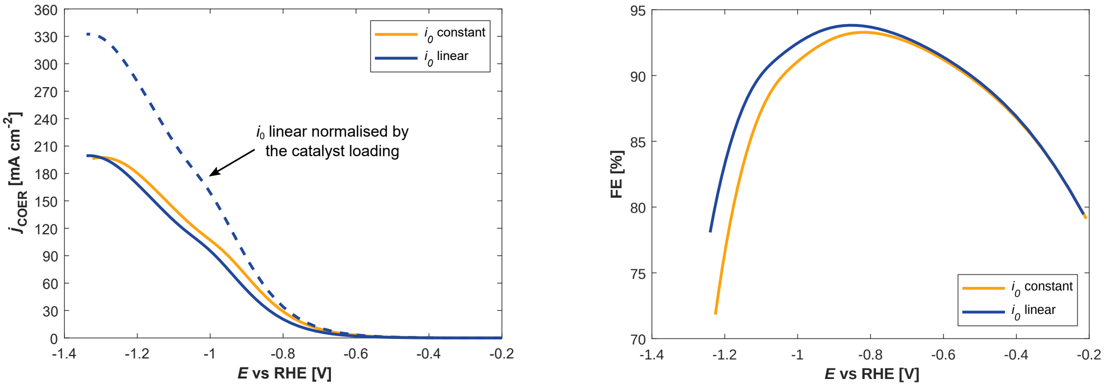

(a) CO_2ER current density against potential for constant catalyst loading and linearly decreasing catalyst loading.

(b) Faradaic efficiency against potential for constant catalyst loading and linearly decreasing catalyst loading.

Figure S7: (a) shows the negligible loss of CO_2ER current density in the linearly reducing catalyst loading case. Given that the linear catalyst loading uses 40% less catalyst, we can normalise the CO_2ER current density by total catalyst used. This normalised current density far exceeds that of the constant catalyst loading. (b) shows that the improvement of the linearly decreasing catalyst loading becomes larger with potential as the reactant depletion becomes the limiting factor.

4 Parameters

Parameter	Description	Value	Unit	Ref.
**Operating parameters**				
p_{abs}	Gas pressure	1	atm	-
T	Temperature	293.15	K	-
c_{KHCO_3}	Catholyte concentration	500	mM	-
Re	Reynolds number	200	-	-
**Geometry**				
L	Flow channel length	20	mm	-
W_{EL}	Flow channel thickness	0.254	mm	-
W_{CL}	CL thickness	3.5	µm	[2]
W_{GDL}	GDL thickness	325	µm	[8]
W_{GAS}	Gas channel thickness	3	mm	-
H	Flow channel height	5	mm	-
**Electrolyte Properties**				
ρ_l	Electrolyte density	998	kg m^{-3}	[9]
μ_l	Electrolyte dynamic viscosity	1×10^{-3}	Pa s	[9]
$D_{\text{CO}_{2,(\text{aq})}}$	CO_2 diffusion coefficient	1.910×10^{-9}	$\text{m}^2\,\text{s}^{-1}$	[2]
$D_{\text{HCO}_3{}^-}$	HCO_3^- diffusion coefficient	1.185×10^{-9}	$\text{m}^2\,\text{s}^{-1}$	[2]
$D_{\text{CO}_3{}^{2-}}$	CO_3^{2-} diffusion coefficient	9.230×10^{-9}	$\text{m}^2\,\text{s}^{-1}$	[2]
D_{OH^-}	OH^- diffusion coefficient	5.293×10^{-9}	$\text{m}^2\,\text{s}^{-1}$	[2]
D_{H^+}	H^+ diffusion coefficient	9.311×10^{-9}	$\text{m}^2\,\text{s}^{-1}$	[2]
**CL Properties**				
ϵ_{CL}	CL porosity	0.5	-	-
a_{v}	CL volumetric surface area	3.75×10^{7}	m^{-1}	[2]
$\sigma_{s,\text{CL}}$	CL conductivity	25	S m^{-1}	[10]
r_{np}	CL average pore radius	4×10^{-8}	m^{-1}	[2]
k_{CO_2}	MPL-CL CO_2 mass transfer coefficient	1.56×10^{-2}	m s^{-1}	[10]
**GDL Properties**				
ϵ_{GDL}	GDL porosity	0.53	-	[8]
σ_{GDL}	GDL conductivity	220	S m^{-1}	[2]
κ	GDL permeability	1.72×10^{-11}	m^2	[8]
λ	GDL average pore radius	2×10^{-6}	m	[10]
**Gas Properties**				
$H_{\text{CO}_2,\text{ref}}$	Henry's constant for CO_2 in water	3.4×10^{-4}	$\text{mol m}^{-3}\,\text{Pa}^{-1}$	[11]
ρ_g	Gas density	1.839	kg m^{-3}	[12]
μ_g	Gas dynamic viscosity	1.469×10^{-5}	Pa s	[12]
$D_{\text{CO}_2,\text{CO}}$	CO_2/CO binary diffusion coefficient	1.52×10^{-5}	$\text{m}^2\,\text{s}^{-1}$	[2]
$D_{\text{CO}_2,\text{H}_2}$	CO_2/H_2 binary diffusion coefficient	6.46×10^{-5}	$\text{m}^2\,\text{s}^{-1}$	[2]
D_{CO,H_2}	CO/H_2 binary diffusion coefficient	7.43×10^{-5}	$\text{m}^2\,\text{s}^{-1}$	[2]
$D_{\text{CO}_2,\text{N}_2}$	CO_2/N_2 binary diffusion coefficient	1.65×10^{-5}	$\text{m}^2\,\text{s}^{-1}$	[2]
D_{CO,N_2}	CO/N_2 binary diffusion coefficient	2.02×10^{-5}	$\text{m}^2\,\text{s}^{-1}$	[2]
$D_{\text{H}_2,\text{N}_2}$	H_2/N_2 binary diffusion coefficient	7.79×10^{-5}	$\text{m}^2\,\text{s}^{-1}$	[2]
**Electrochemistry**				
E^0_{COER}	CO_2 electroreduction potential	-0.11	V	[13]
$i_{0,\text{COER}}$	COER exchange current density	4.71×10^{-4}	mA cm^{-2}	[2]
α_{COER}	COER charge transfer coefficient	0.44	-	[2]
E^0_{HER}	HER electroreduction potential	0	V	[13]
$i_{0,\text{HER}}$	HER exchange current density	1.16×10^{-6}	mA cm^{-2}	[2]
α_{HER}	HER charge transfer coefficient	0.36	-	[2]
E^0_{OER}	OER electroreduction potential	1.23	V	[14]
$i_{0,\text{OER}}$	OER exchange current density	6.21×10^{-5}	mA cm^{-2}	[14]
α_{OER}	OER charge transfer coefficient (for simplified fitted reaction kinetics)	1.5	-	[14]

Table S2: Full list of model parameters

5 Numerical methods

The computational model was solved in COMSOL Multiphysics 5.6, using coupled transport of diluted species and secondary current distribution physics interfaces in the liquid phase, secondary current distribution in the solid phase, and coupled Darcy flow and transport of concentrated species in the gas phase. Dissolved species concentrations in the liquid phase are replaced with logarithmic variables, through $c_i = e^{C_i} \iff C_i = \log(c_i)$, to ensure positivity. Element sizes are scaled by computational region, with priority given to thin regions with high gradients, namely the CL and electrolyte channel, with element density increasing near interfaces and boundaries. Element volumes (2D) range from 10^{-11} m^2 in and around the CL to 10^{-8} m^2 in the gas channel. A preliminary stationary solver was ramped from open circuit potential up to the working potential in the inlet subcell, which was then used as the initial conditions for the full cell model.

References

[1] FP Incropera and DP Dewitt. Fundamentals of heat and mass transfer, john wiley & sons inc. *New-york, p A5 A*, 7, 1990.

[2] Lien-Chun Weng, Alexis T. Bell, and Adam Z. Weber. Modeling gas-diffusion electrodes for CO_2 reduction. *Phys. Chem. Chem. Phys.*, 20:16973–16984, 2018.

[3] Ali Belhocine and Wan Zaidi Wan Omar. Similarity solution and Runge Kutta method to a thermal boundary layer model at the entrance region of a circular tube. *World Journal of Engineering*, 10:1–10, 09 2018.

[4] André Lévêque. *Les lois de la transmission de chaleur par convection.* PhD thesis, Faculté des Sciences de Paris, 1928.

[5] Tiras Y. Lin, Sarah E. Baker, Eric B. Duoss, and Victor A. Beck. Analysis of the reactive CO_2 surface flux in electrocatalytic aqueous flow reactors. *Industrial & Engineering Chemistry Research*, 60(31):11824–11833, 2021.

[6] J.W. Blake, J.T. Padding, and J.W. Haverkort. Analytical modelling of CO_2 reduction in gas-diffusion electrode catalyst layers. *Electrochimica Acta*, 393:138987, 2021.

[7] S. Weisenberger and A. Schumpe. Estimation of gas solubilities in salt solutions at temperatures from 273 K to 363 K. *Aiche Journal*, 42:298–300, 1996.

[8] Ahmad El-kharouf, Thomas J. Mason, Dan J.L. Brett, and Bruno G. Pollet. Ex-situ characterisation of gas diffusion layers for proton exchange membrane fuel cells. *Journal of Power Sources*, 218:393–404, 2012.

[9] https://webbook.nist.gov/cgi/cbook.cgi?ID=C7732185. NIST Chemistry WebBook, Water.

[10] Recep Kas, Andrew G. Star, Kailun Yang, Tim Van Cleve, Kenneth C. Neyerlin, and Wilson A. Smith. Along the channel gradients impact on the spatioactivity of gas diffusion electrodes at high conversions during CO_2 electroreduction. *ACS Sustainable Chemistry & Engineering*, 9(3):1286–1296, 2021.

[11] R. Sander. Compilation of Henry's law constants (version 4.0) for water as solvent. *Atmospheric Chemistry and Physics*, 15(8):4399–4981, 2015.

[12] https://webbook.nist.gov/cgi/cbook.cgi?ID=124-38-9;. NIST Chemistry WebBook, Carbon dioxide.

[13] Y. Hori. Electrochemical CO_2 reduction on metal electrodes. *Modern Aspects of Electrochemistry*, pages 89–189, 2008.

[14] Lien-Chun Weng, Alexis T. Bell, and Adam Z. Weber. Towards membrane-electrode assembly systems for CO_2 reduction: a modeling study. *Energy Environ. Sci.*, 12:1950–1968, 2019.
